# Hantavirus Pulmonary Syndrome Outbreak Anticipation by a Rapid Synchronous Increase in Rodent Abundance in the Northwestern Argentina Endemic Region: Towards an Early Warning System for Disease Based on Climate and Rodent Surveillance Data

**DOI:** 10.3390/pathogens13090753

**Published:** 2024-09-02

**Authors:** Ignacio Ferro, Walter Lopez, Flavia Cassinelli, Sara Aguirre, Griet A. E. Cuyckens, Sebastián Kehl, Daira Abán-Moreyra, Paola Castillo, Carla Bellomo, José Gil, Valeria P. Martinez

**Affiliations:** 1 Andean Ecoregions Institute (Instituto de Ecorregiones Andinas-INECOA), National Scientific and Technical Research Council (CONICET), National University of Jujuy (UNJu), San Salvador de Jujuy 4600, Argentina; flacassinelli@gmail.com (F.C.); grietcuyckens@yahoo.com (G.A.E.C.); 2 Institute for Tropical Disease Research (Instituto de Investigaciones de Enfermedades Tropicales-IIET), National University of Salta (UNSa), Orán A4530, Argentina; wlopezbio@gmail.com (W.L.); jgil@conicet.gob.ar (J.G.); 3 Institute for Non-Conventional Energy Research (Instituto de Investigaciones en Energía No Convencional-INENCO), National Scientific and Technical Research Council (CONICET), National University of Salta (UNSa), Salta A4400, Argentina; sa.agguirre@gmail.com (S.A.); dnabanmoreyra@gmail.com (D.A.-M.); castillopaom@gmail.com (P.C.); 4 National Institute of Infectious Diseases (Instituto Nacional de Enfermedades Infecciosas-INEI), National Administration of Laboratories and Health Institutes (Administración Nacional de Laboratorios e Institutos de Salud-ANLIS “Dr. C. G. Malbrán”), Buenos Aires C1282 AFF, Argentina; skehl@anlis.gob.ar (S.K.); cbellomo@anlis.gob.ar (C.B.); pmartinez@anlis.gob.ar (V.P.M.)

**Keywords:** bunyaviricetes, *Elliovirales*, hantaviridae, orthohantavirus, ecology, epidemiology, rainfall, reservoir

## Abstract

Hantavirus pulmonary syndrome (HPS) is an American emerging disease caused by the rodent-borne virus genus *Orthohantavirus* (Family: *Hantaviridae*: Order: *Elliovirales* Class: *Bunyaviricetes*). In Argentina, almost half of the HPS infections occur in the northwestern endemic region. In this study, we monitored rodent abundance during 2022 and 2023 in three sites with different sampling methods (removal trapping, live trapping and hunted rodents by domestic cats) to evaluate their relationship with human infections. We found a similar pattern of variation in rodent abundance across time, and particularly a synchronous rise of rodent abundance that anticipated an HPS outbreak in 2023. Our dynamic regression models revealed a positive relationship between HPS cases and rodent abundance with a three-month lag, as well as rainfall with an eight-month lag. Our results provide a framework for the planning and implementation of public health prevention campaigns based on climatology and rodent monitoring. Domestic cats bringing rodents into houses can be an overlooked risk factor, particularly if viral shedding of infected rodents is magnified by stress. HPS is a disease of public health concern due to its high mortality rate, the lack of a specific therapeutic treatment and no vaccine. Thus, prevention of infections is of the utmost importance.

## 1. Introduction

Hantavirus pulmonary syndrome (HPS) and hemorrhagic fever with renal syndrome (HFRS) are two emerging zoonotic diseases caused by viruses of Genus *Orthohantavirus.* They are transmitted by wild rodent species of the subfamilies Neotominae and Sigmodontinae in the Americas (HPS) and Arvicolinae and Murinae in Europe and Asia [1]. Human infections occur mainly by inhalation of viral particles in aerosolized excreta from infected rodents. However, some cases of human-to-human transmission have been demonstrated only in Argentina and Chile [2,3]. The virus (*Orthohantavirus*) targets endothelial cells, which produce an inflammatory immunological reaction resulting in increased vascular permeability. The American pathogenic orthohantaviruses species produces pulmonary leakage, edema and cardio-respiratory failure with a fatal outcome of up to 40%, depending on the endemic region and viral genotype [4,5]. The symptomatology appears from 7 to up to 49 days after infection. The prodromic phase, about six days long, is similar to other viral prodromes: high fever, myalgia, nausea, headache, abdominal pain, vomiting and eventually diarrhea. Then, the pulmonary phase begins with dry cough and dyspnea, with mild to moderate respiratory compromise, pulmonary infiltration and pleural effusion that can suddenly evolve to severe respiratory failure with hemodynamic compromise. There is no specific antiviral treatment available nor approved vaccine, and the therapeutic is limited to supportive care in hospitalized patients [6,7]. Thus, prevention of infections is of the utmost importance to protecting the human population. The epidemiological surveillance and the identification of risky factors, areas and time periods are central for reinforcing educational and preventive actions aimed at mitigating human infections and protecting the vulnerable human population.

As a zoonotic infectious disease, HPS outbreaks are often related to environmental changes, presumably affecting the reservoir population’s abundance. Indeed, in North America, the HPS outbreaks have been linked to years of high rainfall induced by an El Niño event, which produces an increase in plant productivity that supports high reservoir density and the subsequent increase of infections in the human population [8,9,10]. Nonetheless, the prediction of zoonotic disease outbreaks is extremely complex, as multiple factors interact at different levels of integration from the pathogen through the host and human social dynamics to trigger an epidemic. This “One Health” approach highlights the complexity of human health, rather than being an isolated phenomenon, as a holistic biospheric process on Earth with interactions that occur between its biological and physical components, including the function of the ecosystems. An important guideline proposed by the “One Health” approach is epidemiological surveillance of pathogens in wildlife. This surveillance allows us to study the occurrence and transmission of infectious agents in wild environments, aimed at preventing and anticipating pathogen emergence. Hitherto, rodent host abundance is a key factor lying at the base of the HPS epidemiological chain, since high reservoir abundance enhances chances of rodent interactions, viral spread into susceptible individuals and contact with the human population. This highlights the importance of rodent surveillance programs to build more accurate predictive models of possible HPS outbreaks. However, this is a challenging task beyond the arduous fieldwork needed to obtain regular longitudinal data collection of temporal variations in reservoir abundance. Rodents have a short generation time of 1.6 years on average, a short gestation period of approximately 60 days, with a mean of five individuals per litter, and a reduced home range [11,12]; populations are susceptible to abrupt numerical fluctuations in short periods of time and in rather localized areas, stochastically influenced by environmental and ecological factors shaping reproduction and survival. Thus, local rodent surveillance generalization based on spatial and temporal scales is blurred by the extremely variable reservoir density.

In Argentina, HPS is a serious concern, rather than due to the number of cases, which averages 70 reported cases yearly, because the vast majority of them required hospitalization (94%); more than 84% developed severe respiratory failure, requiring mechanical ventilation (30%), and there was an overall case fatality rate of 21.4% [6]. There are four endemic regions in the country. The fatality rates increased in south, northeast (13%), northwest (17%) and central (27%) Argentina, and Patagonia (39%); and there is an inverse pattern for HPS prevalence, with almost 50% of all registered cases occurring in northwest Argentina’s endemic region [6,13]. There are two species of pathogenic orthohantaviruses circulating in Argentina, *Orthohantavirus andesense*, represented by Andes virus (ANDV) and *O. negraense* by Laguna Negra virus (LNV) [14]. The main causative etiological agent of HPS in Argentina is the Andes virus (ANDV), with several genotypes in the four endemic regions: Orán virus (ORNV) and Bermejo virus (BERV) in the northwestern Argentina endemic region; Juquitiba virus (JUQV) and Lechiguanas virus (LECV) in the northeastern Argentina endemic region; Central-Plata virus (CPV), Buenos Aires virus (BAV) and LECV in the central Argentina endemic region; and Andes virus (ANDV) in the southern Argentina endemic region [13]. In our studied region, three orthohantaviruses have been identified: Laguna Negra virus (LNV), hosted by *Calomys fecundus*; and two variants of Andes virus (ANDV), Oran (ORNV) and Bermejo (BEMV) virus, carried by *Oligoryzomys chacoensis* and *Oligoryzomys flavescens occidentalis*, respectively [1,6].

Studies on environmental variables that can be used as an indicator of the risk of hantavirus transmission in the country found that precipitation in the warmest season, vegetation cover, evapotranspiration and human demographics were the most important explanatory variables in the transmission of orthohantavirus [15,16,17]. Regarding reservoir studies on a temporal scale, a greater abundance of rodents was associated with a warm and rainy climate in both central and southern endemic regions of Argentina [18,19]. In the northwestern endemic region, we found a significant association between HPS cases with precipitation and temperature from the previous two to six months, valuable as a starting point for the forecast of potential HPS outbreaks based on climatic parameters [20]. Furthermore, we identified temperature, rainfall and vegetation cover as the most important variables for reservoirs in an ecological niche model analysis [21]. However, the linkage between rodent abundance and human infections in northwestern Argentina remains unknown, since there are no available data on rodent temporal variation.

In this report, we explored rodent abundance variation during 2022–2023 across the northwestern Argentina HPS endemic region, in the northern Yungas forest. We aim to assess the temporal and spatial variation of rodent abundance, to fill the knowledge gap on the relationship between rainfall and rodent abundance with HPS incidence, as a step forward for the construction of an early warning system for HPS.

## 2. Materials and Methods

### 2.1. Study Area

**Figure 1:**
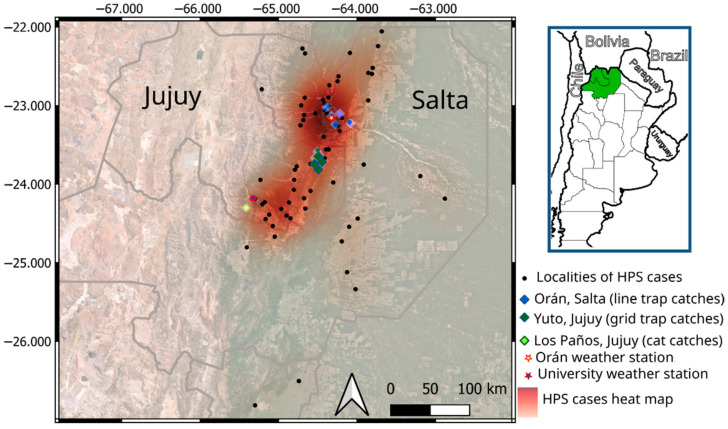
Heat map of accumulated (1997–2017) hantavirus pulmonary syndrome (HPS) cases in the northwestern Argentina endemic region. Black dots are localities of HPS occurrence. Blue rhombuses are rodent sampling localities in Orán, Salta province. Dark green rhombuses are rodent sampling localities in Yuto, Jujuy province. The light green rhombus is the rodent sampling locality in Los Paños, Jujuy province. Stars are the meteorological stations. Scale bar is 100 km.

The northwestern Argentina region encompasses six provinces (Salta, Jujuy, Tucumán, Santiago del Estero, Catamarca and La Rioja) in South America, with very high topographic and environmental variation. However, HPS cases only occur yearly in the foothills and lowlands of northern Salta and eastern Jujuy provinces. Furthermore, in Jujuy province there are 16 departments (third administrative level) but HPS cases only occur in the 6 eastern departments, particularly in Ledemsa department, where near 70% of all cases in the province occur (17% of all HPS cases in the endemic area). Similarly, in Salta province, there are 24 departments (third administrative level) but HPS cases only occur in the 8 northeastern departments, particularly in Orán department where more than 70% of all cases in the province occur (near 57% of all HPS cases in the endemic region) (Figure 1).

We focused on three localities across the latitudinal gradient of the endemic area. Two of them were established where the highest rate of HPS cases occurs, in northern Salta province and northeastern Jujuy province. The third locality was at the southern edge of the endemic region, in Los Paños, a rural place near the capital city of Jujuy province. Most sampling in the northernmost study site was carried out in the surroundings of San Ramón de la Nueva Orán city (23°08′10″ S, 64°19′20″ O, 440 masl) in Orán department and in a few localities of the General José de San Martín, Salta province (22°28′40″ S, 63°48′22″ O, 535 masl). San Ramón de la Nueva Orán is the head city of the Orán department, with a population of 86,000 people, representing half of the department’s inhabitants. All urban locations in this department are surrounded by crops, particularly sugar cane plantations, or degraded (secondary) native forest in the ecotone between the ecoregions of the Yungas Rain Forest and the Chaco semi-arid woodlands. About 70 km south, we established another study site in Jujuy province, around the town of Yuto. This is a small agro-forestry town of 10,000 inhabitants in the north of Ledesma department, Jujuy province (23°39’16” S, 64°28’12” W, 400 masl). Finally, the southernmost study area is 150 km southwards and 1200m above, in the upper mountain forest of a rural place named Los Paños (24°17’04” S, 65°24’06” W, 1600 masl), located 20 km west of San Salvador de Jujuy, the capital city of Jujuy province.

### 2.2. Rodent Abundance Estimates

We compiled data on rodent abundance variation during 2022 and 2023 from different sources in the three sampling areas. The sampling protocol in each studied site was designed for different purposes, to answer specific questions about epidemiology, virus–host dynamics and cat feeding ecology.

### 2.3. Orán Rodent Sampling

In this area, we conducted a rodent removal survey using trap transects in secondary forest at the margin of cornfields, irrigation channels, rivers and peri-urban roads where HPS cases occurred (Figure 1, Table 1). Since this sampling design aimed to assess viral identity and rodent composition in relation to human settings and cases of HPS, rodent sampling efforts and locations were variable. Only one site was sampled twice during 2023 (Hipólito Yrigoyen) before and after the sugar cane harvest. The trap lines remained active for three consecutive nights, baited with rolled oats and re-baited every afternoon. Rodents were euthanized by an overdose of isoflurane inhalation (USP, Ineltano VET, Richmond) and identified to the species level based on external morphology; we recorded total body length, tail length, ear length, weight and sex. Blood samples and tissues were conserved in an ultra-freezer at −80 °C; specimens were fixed in 10% formaldehyde and preserved in 70% alcohol.

### 2.4. Yuto Rodent Sampling

In this area, we conducted standardized rodent sampling aimed at quantifying variations in species abundance and composition across time in different land-use settings. Thus, we used a fixed number of traps (200 traps for three days) every two months in four sites: 1—primary piedmont forest (primary forest), 2—Zausalito, secondary forest, (modified by intensive tree extraction and recurrent fire events), 3—crop fields (fruit trees) and 4—a fruit-packing warehouse situated in Finca los Tucanes surrounded by covered tomato plantations (Figure 1, Table 1). Traps remained active for three consecutive nights, baited with a mixture of peanut, fat and rolled oats, and were re-baited with oats during the second morning. The rodents were anesthetized by inhalation of isoflurane (USP, Ineltano VET, Richmond). Then, we identified every individual to the species level based on external morphology and recorded total body length, tail length, weight and sex; then, the animals were marked and released.

### 2.5. Los Paños Rodent Sampling

In this location, we counted the number of rodents hunted by cats to evaluate the effect of domestic cats as predators of native fauna. Cats are natural predators that will hunt a prey item if they encounter it [22,23,24,25,26,27]. Even when house-based cats are fed and provided with shelter, when free-ranging, pet cats frequently kill wild animals without consuming them and bring them to their owners, allowing for research on hunting habits [28,29,30]. Thus, we estimated rodent abundance by the prey-brought-home method of two free-ranging domestic cats, “Llorisquina” and “Branca”, living in the same house. We monitored every cat’s predation event for 20 months, from March 2022 to December 2023. We recorded the date of every hunting event and photographed prey items for species identification and grouped the hunting events monthly. This area differs from those described above in two important respects. One difference is the location. It is at 1600 m in the Upper Yungas Montane Forest, close to the elevational margin of the HPS endemic area. The other difference is the rodent abundance estimation, which is based on two domestic cats hunting rodents brought into the house and recorded by the cat’s owner instead of trapping sessions.

### 2.6. HPS Cases

HPS is a reportable disease. Thus, we compiled cases of HPS that occurred during 2022–2023 in the northwestern Argentina endemic region (Salta and Jujuy provinces) from the National Health Surveillance System (SISA). We included all cases that were confirmed by laboratory tests with the presence of both IgM and IgG antibodies and/or by viral RNA detection by reverse transcription-quantitative polymerase chain reaction (RTqPCR) and/or RT-PCR followed by nucleotide sequencing. Data reported by the National Health Surveillance System are completely anonymous. Additionally, the National Institute for Infectious Diseases “Instituto Nacional de Enfermedades Infecciosas” (INEI) and the National Health Surveillance System (SISA) follow the principles of ethics in research for the collection of data keeping with the Declaration of Helsinki on human study and the Research in Human Beings Guide of the National Ministry of Health.

### 2.7. Climatic Variables

We obtained climatic data from the Meteorological Information Center of the National Meteorological Service, Argentina, within the public data policy (EXP 200,807). We compiled daily precipitation in mm of rainfall accumulated during 24 h since 9am from two weather stations during 2022 and 2023. The weather station is at the airport of Oran city, representing Yuto and Orán rainfall climatology at the core of the northwestern Argentina endemic region (Figure 1).

### 2.8. Data Analysis

To compare the data on rodent abundance collected by trapping sessions with different sampling efforts, we established the minimum trap-night effort (150 trap nights) as a baseline to refer to trap success in localities with higher trapping effort (number of trapped rodents/trapping effort*150). However, the estimated rodent abundance by means of the prey-brought-home method is the monthly number of rodents caught by two hunting cats. Similarly, the monthly number of HPS cases and the accumulated millimeters of rainfall every month are measured on different scales. Thus, we standardized all variable magnitudes on the same scale using the number of standard deviations, Z-score, for further comparisons. We performed all the analyses using R Statistical Software v4.1.2; R Core Team 2021/11/01.

To assess the relationship of rodent abundance variation across time, we visualized the plotted data from the three studied sites across time and then used pairwise Spearman rank correlation coefficient between matching time data for the different studied sites. We used the linear interpolation function in the xts package in R to handle missing data. We evaluated stationary data using the augmented Dickey–Fuller test in the tseries package [31]. Then, we analyzed the lagged effect of rodent abundance and rainfall on HPS through an exhaustive search for eight lagged months and five autoregressive (AR) and moving average (MA) components in the error term using the forecast packages [32]. We ranked the best candidate models by minimizing the Akaike information criterion corrected for small samples (AICc) [33]. Finally, we checked for the random residuals distribution of the selected models using the Ljung–Box test.

## 3. Results

### 3.1. Rodent Sampling

We obtained 44 rodent record events during the two-year monitoring period in the three sites (Table 1). The case-driven trapping sessions in Orán, Salta Province were the most heterogeneous, both spatially and temporally, with 10 trapping events in 8 sites and a total of 404 captured individuals belonging to nine rodent species. The most frequent species was *Calomys fecundus* (56.8%) followed by *Akodon simulator* (23.1%), *Oligoryzomys chacoensis* (7.5%), *O. flavescens occidentalis* (6.9%), *Mus musculus* (2.7%), *Holochilus chacarius* (1.3%), *O. brendae* (0.7%), *Calomys musculinusus* (0.3%) and *Rattus ratus* (0.3%).

In Yuto, the standardized rodent trapping returned 12 sampling events, regularly spaced every two months in spatially fixed sampling sites, with a total of 168 captured individuals of six species. *Akodon simulator* (52%) and *Calomys fecundus* (38%) were the most frequently trapped species, followed by *Euryoryzomys legatus* (4%), *Oligoryzomys chacoensis* (3%), *C. musculinus* (2%) and *Rattus rattus* (1%).

In Los Paños, we counted a total of 51 rodents hunted by the two cats from March 2022 to December 2023, of which 4 could not be identified at any taxonomic level, and 3 were identified only at the generic level. For the remaining 44 individuals, we recorded five species, of which *Calomys fecundus* (30%) and *Akodon caenosus* (27%) were the most frequent, followed by *Oligoryzomys flavescens occidentalis* (20%), *A. fumeus* (18%) and *O. brendae* (5%).

**Figure 2:**
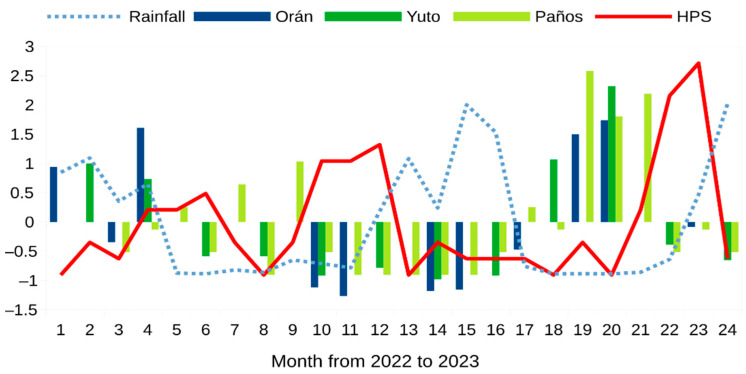
Standardized rainfall (dotted light blue line) rodent abundance (blue bars: rodent survey in Orán, Salta province; dark green bars: Yuto rodent survey in Jujuy province; light green bars: Los Paños town in Jujuy province) and hantavirus pulmonary syndrome (red line) during the 2-year study period.

The temporal variation in rodent abundance was similar despite sampling sites and methods, as shown by standardized abundance across time (Figure 2). The pairwise comparison of matching time events between sites showed a significant positive correlation. The Spearman rank correlation coefficient for the 10 matching time events between Orán and Paños surveys was r_s_ = 0.83, *p*-value = 0.002. For Yuto and Orán, the five matching events also revealed a positive correlation of r_s_ = 0.91, *p*-value = 0.04. Similarly, the correlation between Paños and Yuto rodent abundance for the 11 time-matching survey events was positive and significant r_s_ = 0.79, *p*-value = 0.003.

### 3.2. Human Orthohantavirus Infection

We recorded 78 confirmed cases of HPS in the northwestern Argentina endemic region during 2022 and 2023, with a total of 42 human infections during 2022 and 36 in 2023. The temporal distribution of infections was somewhat variable between these two years. During 2022, infections were more evenly distributed, with two moderate peaks in autumn and spring, while in 2023 there was a single outbreak with almost all cases concentrated in October and November during the early southern spring (Figure 2).

### 3.3. Orthohantavirus Human Infections and the Lagged Rodent Abundance

**Figure 3:**
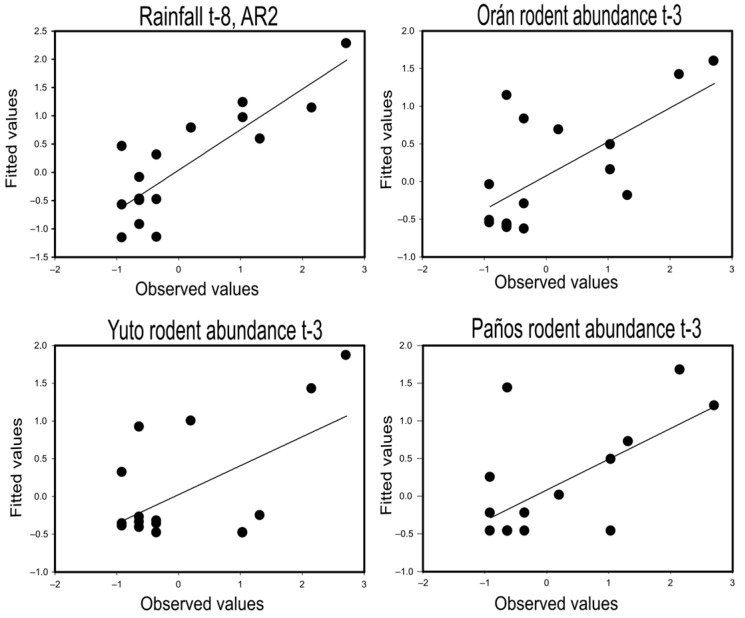
Plots of observed hantavirus pulmonary syndrome cases vs. fitted values by the selected models for rainfall with eight-month lag (t-8) and rodent abundance with three-month lag (t-3) in Orán, Salta province; Yuto Jujuy province; and Paños in Jujuy province as explanatory variables.

The time series data were stationary according to the augmented Dickey–Fuller test (ADF = −3.63, *p*-value = 0.04). The results of model selection for HPS cases related to the delayed rainfall and the rodent abundance in each studied site are listed in Table 2. For lagged rainfall as an explanatory variable, we found the most parsimonious significant model with the precipitation 8 months before, either with a second-order autoregressive AR2 or moving average MA2 process in the error term (Table 2). This model revealed a significant positive relationship between HPS cases (Figure 3) and left no significant structure remaining in model residuals according to the Ljung–Box test (Table 2 and Table 3).

For the delayed rodent abundance as an explanatory variable of HPS cases, we found a three-month lagged time for all the rodent sampling sites (Table 2). Considering the rodent trap-success data in Orán, we found a model with three lagged periods (AIC = 47.5) that accounted for 41% of HPS cases variation with no autoregressive nor moving average needed to account for serial dependence (Table 2, Figure 3). Additionally, we found a model with four lagged months (AIC = 48.6) and a first-order moving average MA1 in the error term that accounted for 51% of HPS cases (Table 2). For the lagged rodent abundance in Yuto, we found a single best model with three lagged months (AIC = 48.6) that accounted for 36% of the variation in HPS cases (Table 2, Figure 3). Similarly, for the number of rodents hunted by cats in Los Paños, we found a three-month lagged model (AIC = 48.6) that accounted for 36% of the HPS variation and a two-month lagged model (AIC = 49.2) that accounted for 34% of the HPS cases variation. All models revealed a positive relationship between lagged rodent abundance and rainfall with orthohantavirus infections (Table 3, Figure 3).

## 4. Discussion

In this paper, we investigated the relationship between HPS cases with lagged rainfall and rodent abundance, searching for signals that may serve as early warning of potential HPS outbreaks in the northwestern Argentina endemic area. Our analysis indicates that both the rainfall and rodent abundance have a lagged positive relationship with HPS cases of eight and three months, respectively. For this endemic area, we have already reported a positive relationship of lagged rainfall with HPS cases [20]. However, there are no available data on reservoir abundance variation for this endemic area, which represents the main contribution of the present study. The assumed role of rainfall in HPS outbreaks is in its effect on vegetation growth, the primary production of an ecosystem, which raises its carrying capacity and facilitates rodent survival through food input and shelter [9,34,35]. Our results provide some support for this hypothesis by the sequential lagged time for each variable (Table 2). Rainfall correlated positively with HPS cases eight months before and rodent abundance three months before the rise in HPS cases.

We recorded temporal variations in rodent abundance in three different areas of the northwestern Argentina HPS endemic region during 2022 and 2023. Though our source of information to estimate the rodent’s abundance was not homogeneous, considering either sampling protocols, spatial replication or temporal spacing, we found a similar pattern of variation across time (Figure 2) as revealed by pairwise correlation analysis of matching time sampling events. This similar pattern of variation across time and space suggests that the rodent assemblage responds to regional environmental conditions, which may allow us to generalize sampling points to a larger part of the endemic area. However, we must raise a flag of caution. Our two-year study represents a very short time lapse to draw strong conclusions. The HPS outbreak at the end of 2023 was clearly preceded by a rapid and synchronous rise in rodent abundance in the three studied sites (Figure 2). However, the rodent sampling was more scattered in 2022. There is evidence of a parallel rise in rodent abundance in Yuto and Oran, the core northwestern Argentina HPS endemic region, prior to the 2022 autumn peak of orthohantavirus infections. Unfortunately, we missed the Los Paños rodent abundance, since we started to count hunted rodents in March 2022. The inverse situation occurred for the second HPS peak in the spring of 2022. We have no samplings from winter in Oran. Additionally, a huge forest fire during the winter in 2022 affected large areas of northern Salta and Jujuy provinces. In particular, our study site in Yuto was burned almost completely [36]. Thus, we have no captures during the second half of 2022 in the barren Yuto secondary forest. This configured a rodent abundance data silence for the hot-spot endemic area (Yuto and Oran) previous to the HPS peak in spring 2022, but not for Los Paños where there seems to be a rodent abundance rise prior to the HPS peak that year (Table 1, Figure 2). The localized effects of stochastic events, such as fire, floods, deforestation or habitat selection, as well as depredation and competition, may blur the regional environmental influences on rodent species fitness. Nonetheless, our analysis shows a clear signal of increased rainfall and rodent abundance prior to human infections in the northwestern Argentina HPS endemic area (Table 2, Figure 3).

However, the risk of human infection does not depend solely on rodent abundance but on several levels of interactions from the environmental influence, of which only rainfall was evaluated in this study. Moreover, ecological responses of the rodent community, such as competition and depredation, which were not evaluated here, through the viral transmission dynamics in the specific reservoirs population to human behavior and social practices that promote rodent–human encounters, were also beyond the aim of this study [37,38]. We recorded the whole rodent community variation across time and space in our three study sites. However, the effect of host and non-host species interaction is still far from being clarified. For example, the dilution effect suggests that high assemblage diversity may reduce individual interactions of the host species, reducing the risk of transmission within the reservoir population [39]. The host species contact rate is important to propagate orthohantavirus infections given the horizontal nature of viral transmission in reservoirs, and therefore in HPS epidemiology. On the other hand, spillover has been suggested as a mechanism to maintain viral transmission at a community level, and its role in HPS outbreaks should be deeply assessed [40]. Acknowledging the limitations of our data to fully understand all the levels of interconnections involved in HPS outbreaks, our results suggest that the greater the rodent abundance, the more species present in an assemblage (Table 1). Indeed, there is a positive and significant correlation between rodent abundance and species richness in Orán (r_s_ = 0.76 *p* =0.01 n=10), Yuto (r_s_ = 0.81 *p* = 0.001 N = 12) and Los Paños (r_s_ = 0.79 *p* = 0.001 N = 22). Thus, despite the possible effect of species diversity on host viral transition, either negative or positive feedback, the overall rodent abundance also raises the reservoir species number, increasing the risk of contact with humans and thus orthohantavirus infections. Furthermore, domestic hunting cats do not always eat rodents immediately but bring them into homes, frequently alive, from the surrounding wild areas. This can be an overlooked risk factor that should be further investigated in South America, particularly if viral shedding of infected rodents is magnified by stress. Indeed, cat ownership has been reported as a risk factor for hemorrhagic fever with renal syndrome (HFRS) disease in Asia, suggesting that infected cats may be important in spreading the virus to humans [41]. However, studies in North America and Europe found low infection rates in cats and did not find evidence of persistent infection, suggesting dead-end infections with a low risk of virus excretion, like that which occurs with human infections [42,43,44].

The three-month delay in HPS cases related to rodent abundance may be related to the horizontal transmission of the virus in their reservoirs. Frequently, the prevalence of antibodies in the host rodent population is low during peaks of abundance because the growing population has many juvenile naive individuals who need to interact with infected animals in the population to acquire the virus [45,46,47]. Thus, the prevalence of infections in the population may be higher after the peak of abundance, although with lower absolute numbers of individuals. Additionally, it is likely that a recently infected viremic individual will heavily excrete the virus. To add complexity, a simple increase in rodent abundance does not always trigger outbreaks, because of a density threshold needed to spread the infection in the population [10]. However, our results are valuable as a starting point to build an early warning system for HPS disease based on rodent surveillance. Furthermore, in this region there are several febrile syndromes with similar symptomology that make accurate clinical diagnosis difficult. Since HPS must be differentiated early in the prodromal phase, the prevention campaign should be intensified during the rodent peak of abundance among health systems staff, rural workers and the general population.

## 5. Conclusions

In conclusion, beyond the above-described limitations of this study, we found a relationship between rainfall, rodent dynamics and the number of HPS cases in northwestern Argentina. The rodent abundance dynamics, which were previously unknown in the region, seem to be geographically synchronous and detectable by different methods. These characteristics may facilitate rodent monitoring. The three-month lagged rodent abundance relationship with HPS outbreaks is valuable for planning environmental management practices. Furthermore, we found that HPS outbreaks coincided with two different factors, a rodent abundance growth and rainfall prior to rodent population explosions. This sequential lagged relationship supports the tropic cascade hypothesis, which establishes that rainfall impacts rodent population growth and subsequent human infections. The interactions between these different parameters make it possible to build a two-level tool to anticipate and prevent possible HPS outbreaks, based on rainfall first and rodent abundance later. These findings represent an important step towards the development of early warning tools for possible high risk of infections in humans for this endemic region, which should be refined by further investigations to enhance our knowledge of HPS disease dynamics.

## Figures and Tables

**Table 1:** Monthly numbers of HPS cases in the northwestern Argentina endemic region, rainfall (mm) in Orán airport, rodent abundance for each sampling event and species richness (number of species) in the three studied localities. In Orán and Yuto, trap success is the number of individuals expected in a 150 trap night sampling effort, while in Paños the monthly numbers represent rodents hunted by two domestic cats.

Year	Month	HPS Cases	Orán Rainfall	Orán Trap Success	Orán Species Richness	Yuto Trap Success	Yuto Species Richness	Paños Hunted Rodents	Paños Species Richness
2022	1	0	103.5	26.1	3				
2022	2	2	118.1			7.5	5		
2022	3	1	74.0	11.4	5			1	1
2022	4	4	91.7	33.5	5	6.5	5	2	2
2022	5	4	0.8					3	2
2022	6	5	0.1			1.5	2	1	1
2022	7	2	4.0					4	3
2022	8	0	1.5			1.5	2	0	0
2022	9	2	14.1					5	3
2022	10	7	10.4	2.69	4	0.25	1	1	1
2022	11	7	6.3	1.01	2			0	0
2022	12	8	63.8			0.75	3	0	0
2023	1	0	117.5					0	0
2023	2	2	67.5			0	0	0	0
2023	3	1	172.5	2.26	3			0	0
2023	4	1	144.5			0.25	1	1	3
2023	5	1	7.8	10.02	2			3	2
2023	6	0	0			7.75	3	2	3
2023	7	2	0.1	32.3	8			9	3
2023	8	0	0	35.3	6	12.75	3	7	3
2023	9	4	1.7					8	1
2023	10	11	12.8			2.25	3	2	1
2023	11	13	80.7	14.4	5			2	2
2023	12	1	173.6			1.25	2	1	1

**Table 2:** The selected dynamic regression models for different combinations of lagged rainfall and lagged rodent abundance with ARIMA error, listed according to the minimum Akaike information criterion corrected for small samples (AICc).

Models	Model Selection	AICc	Ljung–Box Test	R^2^adj	*p*-Value
HPS~Lagged Rainfall	Rainfall 8 months before, AR2	47.4	Q* = 1.29, *p*-value = 0.73	0.69	>0.01
	Rainfall 8 months before, MA2	48.1	Q* = 4.12, *p*-value = 0.24	0.71	>0.01
	Rainfall 7 months before	49.2	Q* = 4.14, *p*-value = 0.24	0.34	0.01
	Rainfall 8 months before	49.6	Q* = 5.47, *p*-value = 0.14	0.32	0.01
	Rainfall 8 months before, MA1	50.1	Q* = 1.79, *p*-value = 0.52	0.59	>0.01
HPS~Lagged Orán Rodent Abundance	Trap-success 3 months before	47.5	Q* = 1.42, *p*-value = 0.69	0.41	>0.01
	Trap-success 4 months before, MA1	48.6	Q* = 2.76, *p*-value = 0.43	0.58	>0.01
	Trap-success 3 months before, MA1	50.9	Q* = 1.48, *p*-value = 0.68	0.42	0.01
	Trap-success 4 months before	50.9	Q* = 3.13, *p*-value = 0.37	0.26	0.02
	Trap-success 3 months before, AR1	51	Q* = 1.81, *p*-value = 0.61	0.41	>0.01
HPS~Lagged Yuto Rodent Abundance	Trap-success 3 months before	48.6	Q* = 0.72, *p*-value = 0.86	0.36	>0.01
	Trap-success 4 months before, MA1	51.4	Q* = 0.95, *p*-value = 0.81	0.40	>0.01
	Trap-success 3 months before, AR1	51.6	Q* = 0.99, *p*-value = 0.80	0.39	>0.01
	Trap-success 2 months before	52.1	Q* = 3.01, *p*-value = 0.38	0.16	0.06
	Trap-success 4 months before	52.8	Q* = 1.17, *p*-value = 0.75	0.17	0.06
HPS~Lagged Paños Rodent Abundance	Hunted rodents 3 months before	48.8	Q* = 1.25, *p*-value = 0.73	0.36	>0.01
	Hunted rodents 2 months before	49.2	Q* = 0.72, *p*-value = 0.86	0.34	0.01
	Hunted rodents 3 months before, MA1	51.9	Q* = 1.16, *p*-value = 0.76	0.38	>0.01
	Hunted rodents 3 months before, AR1	52	Q* = 1.44, *p*-value = 0.69	0.37	>0.01
	Hunted rodents 2 months before, MA1	52.6	Q* = 0.70, *p*-value = 0.87	0.34	0.01

**Table 3:** Estimated coefficients for the selected dynamic regression models of standardized z-values of hantavirus pulmonary syndrome cases, the rainfall in Orán, and the estimated rodent abundance in the three studied sites: Orán, Yuto and Los Paños. AR for autoregressive and MA for moving average coefficient of the ARIMA error term component.

Selected Models	Coefficient	Estimated	Standard Error
HPS~Rainfall 8 month before, AR2	Rainfall t-8	0.89	0.13
	AR1	−0.03	0.25
	AR2	−0.81	0.15
HPS~Orán trap-success 3 months before	Rodent Abundance t-3	0.74	0.20
HPS~Yuto trap-success 3 months before	Rodent Abundance t-3	0.69	0.20
HPS~Paños hunted rodents 3 months before	Rodent Abundance t-3	0.61	0.18

## Data Availability

The data presented in this study are all available in the manuscript. However, the corresponding author will provide any additional data upon reasonable request.
